# Upper Airway Sensory Testing in Dysphagia – Implications for Clinical Practice and Future Research Directions

**DOI:** 10.1007/s00455-024-10789-w

**Published:** 2024-12-03

**Authors:** Norita Regio, Ruby Hutton, Emma S. Wallace

**Affiliations:** Discipline of Speech Pathology, Sydney School of Health Sciences, Faculty of Medicine and Health, The University of Sydney, Sydney, Australia

**Keywords:** Sensory testing, Swallow assessment, Upper airway, Sensation, Dysphagia, Systematic review

## Abstract

Adequate upper airway (oral, pharyngeal and laryngeal) sensation is crucial for safe and efficient swallowing and airway protection. Despite its importance, methods of upper airway sensory testing for individuals with dysphagia remain poorly defined. The aim of this study was to summarise and appraise current methods of upper airway sensory testing to inform dysphagia clinical practice and future research directions. A systematic review was conducted using the Preferred Reporting Items for Systematic Reviews and Meta-Analyses guidelines. Studies that reported a method of upper airway sensory testing in individuals with dysphagia were included. Of the 1187 studies identified and screened, 54 met the inclusion criteria. Four types of upper airway sensory testing were reported: Flexible Endoscopic Evaluation of Swallowing with Sensory Testing (*n* = 31), Cough Reflex Testing (*n* = 22), Gag Reflex Testing (*n* = 5) and Gustatory (taste) Testing (*n* = 2). Indeterminate “tests of sensory function” (*n* = 2) were also reported. Variability in methods was evident across studies and reporting was insufficient for reliable replication. This review provides a comprehensive overview of methods of upper airway sensory testing in the dysphagia literature for dysphagia clinicians and researchers. The results indicate a need to develop a valid and reliable upper airway sensory testing protocol for individuals with dysphagia to improve assessment and targeted treatments.

**Supplementary Information:**

The online version contains supplementary material available at 10.1007/s00455-024-10789-w.

## Introduction

Upper airway sensation refers to sensory information from the nasal cavities, oral cavity, pharynx, and larynx. This information is vital to coordinate and modulate the complex sequence of swallowing motor events and regulate airway protective responses, such as the laryngeal adductor reflex (LAR) and cough response [1].

The importance of upper airway sensation is highlighted by numerous studies demonstrating that impaired upper airway sensation, induced by intra-oral, pharyngeal, or laryngeal anaesthesia, resulted in impaired swallowing safety and efficiency [2–6]. In healthy individuals with upper airway anaesthesia, 43% demonstrated laryngeal penetration and of those, half demonstrated tracheal aspiration [5]. This was attributed to impaired closure of the laryngeal vestibule during swallowing, despite participants’ ability to close the larynx during volitional tasks [5]. Similar findings were reported by Sulica and colleagues, where a bilateral superior laryngeal neve block in healthy individuals resulted in premature spillage, pharyngeal residue, laryngeal penetration and tracheal aspiration for both puree and liquid boluses, despite adequate motor function [5]. In individuals with dysphagia, laryngopharyngeal sensory deficits are associated with move severe swallowing impairment and the development of aspiration pneumonia across multiple patient populations [7–10].

Despite its importance, assessment of upper airway sensation presents a challenge in research and clinical contexts. There is little consensus on how upper airway sensation should be assessed in individuals with dysphagia and variations in clinical practice are evident [11, 12]. This limits the ability to identify impaired sensation contributing to swallowing impairments and to develop targeted treatment for impaired upper airway sensation. Therefore, the aim of this study was to summarise and appraise methods of upper airway sensory testing in the dysphagia literature to inform clinical practice and future research directions.

## Methods

### Protocol and Registration

The current study was registered in the international prospective register of systematic review (PROSPERO) on 1st August 2022 (Registration number: CRD42022341953). Adherence to the Preferred Reporting Items for Systematic reviews and Meta-Analyses (PRISMA) was followed when reporting. Methods were informed by “A Measurement Tool to Assess the Methodological Quality of Systematic Reviews (AMSTAR)” guidelines [13].

### Inclusion and Exclusion Criteria

Primary research in adults (> 18 years) with oropharyngeal dysphagia that included an upper airway (oral, pharyngeal, laryngeal) sensory test, were included in the review. Studies on children, languages other than English and animal studies were excluded. Studies on upper airway sensory rehabilitation were excluded unless a measure of upper airway sensory testing was used as an outcome measure. Studies evaluating cortical neuronal activity in response to sensory stimuli via somatosensory evoked potentials were also excluded due to the clinical focus of the review.

### Search Terms

Databases (Medline via Ovid and PubMed) were searched up to July 1st, 2022. Eligible titles and abstracts generated from the database search were selected and inputted into Covidence. The full search strategy was as follows:

1. Deglutition Disorders / deglutition disorder*.tw. / dysphagia.tw. / swallowing disorder*.mp. / silent aspiration.tw.
2. (upper airway* adj4 (Sensation* or Function* or Test* or Physiolog* or Sensory)).tw. /Sensorimotor function*.tw. / sensation/ or proprioception/ or smell/ or taste/ or thermosensing/ or touch/ Vibration/ (propriocept* or smell or taste or gustat* or themo* or hot or cold or touch or vibration*).tw. / Tactile thermal*.tw. / cough test*.tw./ sensory test*.tw. / urge to cough.tw. / somatosen*.tw.
3. 1 and 2.

### Study Selection

Two reviewers (NR, RH) screened all abstracts and full texts for inclusion to reduce the risk of bias of an individual rater, as per AMSTAR guidelines [14]. Any disagreements between reviewers were resolved by discussion, or a third reviewer (EW), to reach consensus.

### Data Extraction

Data was extracted from full texts and recorded on an Excel spreadsheet. All data was checked by two reviewers (NR, EW). The following information was extracted from the studies: dysphagia aetiology, sample size, age, name of sensory test, anatomical location assessed, type of sensation assessed, stimulus, equipment, presentation of stimulus, control stimulus used, procedures or protocols referenced, response observed and the test end point and criteria for normal response.

### Analysis of Risk of Bias and Quality

A risk of bias analysis was not conducted for each study as data was extracted on the study methods only. The quality of the included studies were evaluated against the National Health and Medical Research Council (NHMRC) Designation of Levels of Evidence hierarchy [15].

## Results

**Fig. 1:**
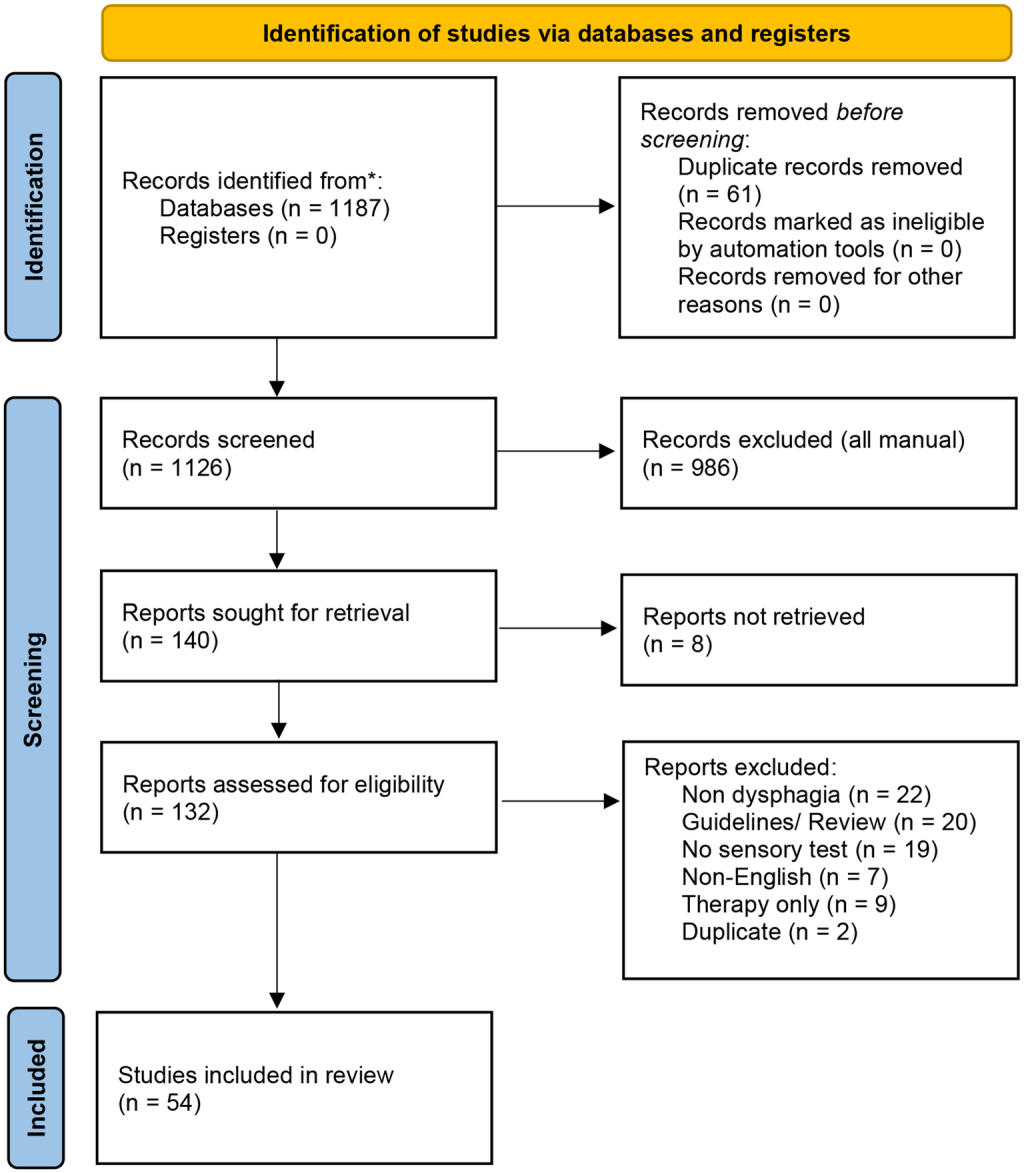
Study selection procedure

1187 papers were retrieved and screened from the electronic databases. On the basis of the exclusion criteria, 54 studies were included in the final review. The study selection procedure is detailed in Fig. 1 [16].

### Characteristics of the Included Studies

Included studies ranged from 1996 to 2022. Studies included individuals with dysphagia secondary to cerebrovascular diseases, neurodegenerative disorders, respiratory disease, head and neck cancer, surgery, gastroesophageal reflux disease, autoimmune disorders, traumatic brain injury, vocal fold paralysis, oesophageal stricture, and prolonged intubation. Included studies comprised cross-sectional studies (66%), cohort studies (17%), case-controlled studies (13%), and case series (4%).

### Methods of Upper Airway Sensory Testing

Four upper airway sensory tests were identified from the included studies: Cough Reflex Testing (CRT) [17–37], Fibreoptic Endoscopic Evaluation of Swallowing with Sensory Testing (FEESST) [7, 17, 18, 38–63], gag reflex testing [11, 17, 18, 57, 64] and taste testing [65, 66]. Two studies included “sensory testing” [11, 64] but did not elaborate on what the tests constituted. Five studies reported two or more methods of upper airway sensory testing within the study [11, 17, 20, 57, 64]. A full summary of the methods of upper airway sensory testing in the dysphagia literature are provided in Table 1.

**Table 1:** Methods of oral, pharyngeal and laryngeal sensory testing in the dysphagia literature

First Author	Year	Nature of Dysphagia	Test name	Type of sensation assessed	Stimulus	Response Observed	Criteria for Normal Response
Giraldo-Cadavid et al. [17]	2016	Mixed aetiologies	CRT	Tactile	Air pulses	NR	NR
Kambayashi et al. [18]	2021	N/A	CRT	Chemical	NR	NR	NR
Yiu et al. [19]	2019	Neurodegenerative disorders	CRT	Chemical	Capsaicin aerosol	Self-rated urge-to-cough (UtC)	Subjective score = 0
Borders et al. [20]	2022	Neurodegenerative disorders	CRT	Chemical	Capsaicin aerosol	Cough (presence or absence) + UtC	Subjective score = 10 on a modified Borg scale
Troche et al. [21]	2014	Neurodegenerative disorders	CRT	Chemical	Capsaicin aerosol	Cough (presence or absence), UtC	Median Urge to Cough (UTC) of 5 at 200 µM
Smith & Wiles [22]	1998	Mixed aetiologies	CRT	Chemical	Capsaicin aerosol	Cough (presence or absence)	NR
Borders et al. [23]	2021	Neurodegenerative disorders	CRT	Chemical	Capsaicin aerosol	Cough (presence or absence) + UtC	NR
Silverman et al. [24]	2016	TBI	CRT	Chemical	Capsaicin aerosol	Cough (presence or absence)	NR
Tabor-Grey et al. [25]	2021	Neurodegenerative disorders	CRT	Chemical	Capsaicin aerosol	Cough (presence or absence) + UtC	NR
Troche et al. [26]	2016	Neurodegenerative disorders	CRT	Chemical	Capsaicin aerosol	Cough (presence or absence) + UtC	NR
Curtis & Troche [27]	2020	Neurodegenerative disorders	CRT	Chemical	Capsaicin aerosol	Cough (presence or absence) + UtC	NR
Wakasugi et al. [28]	2008	Mixed aetiologies	CRT	Chemical	Citric acid aerosol	Cough (presence or absence)	≥ 5 coughs
Sakai et al. [29]	2016	Mixed aetiologies	CRT	Chemical	Citric acid aerosol	Cough (presence or absence)	Cough within 30 s
Wakasugi et al. [30]	2014	Mixed aetiologies	CRT	Chemical	Citric acid aerosol	Cough (presence or absence)	≥ 5 coughs
Guillen-Sola et al. [31]	2015	Cerebrovascular disease	CRT	Chemical	Citric acid aerosol	Cough (presence or absence)	≥ 5 coughs
Lee et al. [32]	2014	Mixed aetiologies	CRT	Chemical	Citric acid aerosol	Cough (presence or absence)	Cough within 28.13 s
Sato et al. [33]	2012	Mixed aetiologies	CRT	Chemical	Citric acid aerosol	Cough (presence or absence)	Cough within 5 s
Sohn et al. [34]	2018	Cerebrovascular disease	CRT	Chemical	Citric acid-sodium chloride aerosol	Cough (presence or absence)	NR
Dallal-York et al. [35]	2023	Surgical Procedure	CRT	Chemical	Citric acid-sodium chloride aerosol	Cough (presence or absence)	Cough within 60 s
Choi et al. [36]	2017	Cerebrovascular disease	CRT	Chemical	Citric acid-sodium chloride aerosol	Cough (presence or absence)	NR
Fujiwara et al. [37]	2017	Mixed aetiologies	CRT	Chemical	Tartaric acid aerosol	Cough (presence or absence)	Cough within 1 s
Ohno et al. [83]	2021	Mixed aetiologies	CRT	Chemical	Tartaric acid aerosol	Cough (presence or absence)	NR
Aviv et al. [38]	1997	Cerebrovascular disease	FEESST	Tactile	Air pulse	LAR	NR
Aviv et al. [39]	1998	Mixed aetiologies	FEESST	Tactile	Air pulse	LAR	LAR at < 4 mmHg air pulse pressure
Cuellar & Harvey [40]	2019	Cerebrovascular disease	FEESST	Tactile	Air pulse	LAR	LAR at < 4 mmHg air pulse pressure
Kaneoka et al. [41]	2015	Mixed aetiologies	FEESST	Tactile	Air pulse	LAR	LAR at < 4 mmHg air pulse pressure
Aviv et al. [42]	2000	NR	FEESST	Tactile	Air pulse	LAR	LAR at < 4 mmHg air pulse pressure
Aviv et al. [7]	1997	Cerebrovascular disease	FEESST	Tactile	Air pulse	LAR	LAR at < 4 mmHg air pulse pressure
Martin et al. [43]	1999	Mixed aetiologies	FEESST	Tactile	Air pulse	LAR	LAR at < 4 mmHg air pulse pressure
Aviv et al. [44]	2001	Mixed aetiologies	FEESST	Tactile	Air pulse	LAR	LAR
Aviv et al. [45]	2001	Head and neck cancer	FEESST	Tactile	Air pulse	LAR	NR
Parise Junior et al. [46]	2004	Mixed aetiologies	FEESST	Tactile	Air pulse	LAR	LAR at < 4 mmHg air pulse pressure
Setzen et al. [47]	2003	NR	FEESST	Tactile	NR	LAR	LAR at < 4 mmHg air pulse pressure
Aviv et al. [48]	2005	Mixed aetiologies	FEESST	Tactile	Air pulse	LAR	LAR at < 4 mmHg air pulse pressure
Amin et al. [49]	2006	Neurodegenerative disorders	FEESST	Tactile	Air pulse	LAR	LAR at < 4 mmHg air pulse pressure
Cohen et al. [50]	2003	Mixed aetiologies	FEESST	Tactile	Air pulse	LAR	NR
Ku et al. [51]	2010	Head and neck cancer	FEESST	Tactile	Air pulse	LAR	LAR at < 4 mmHg air pulse pressure
Sasaki et al. [52]	2006	Surgical Procedure	FEESST	Tactile	Air pulse	LAR	NR
Tabaee et al. [53]	2006	NR	FEESST	Tactile	Air pulse	LAR	NR
Giraldo-Cadavid et al. [17]	2016	Mixed aetiologies	FEESST	Tactile	Air pulse	LAR	NR
Hammer et al. [54]	2016	Surgical Procedure	FEESST	Tactile	Air pulse	LAR	LAR at < 4 mmHg air pulse pressure
Schindler et al. [55]	2010	Head and neck cancer	FEESST	Tactile	Air pulse	LAR	LAR at < 4 mmHg air pulse pressure
Aviv et al. [56]	2002	Mixed aetiologies	FEESST	Tactile	Air pulse	LAR	LAR at < 4 mmHg air pulse pressure
Aviv et al. [57]	2000	NR	FEESST	Tactile	Air pulse	LAR	LAR at < 4 mmHg air pulse pressure
Giraldo-Cadavid et al. [58]	2018	Mixed aetiologies	FEESST	Tactile	Air pulse	NR	NR
Cuellar & Harvey [40]	2019	Cerebrovascular disease	FEESST	Tactile	Tip of endoscope	LAR	LAR
Kambayashi et al. [18]	2021	N/A	FEESST	Tactile	Tip of endoscope	NR	NR
Kaneoka et al. [59]	2018	NR	FEESST	Tactile	Tip of endoscope	Cough (presence or absence), gag reflex, swallow, LAR	Cough, gag, swallow or LAR
Marian et al. [60]	2017	Cerebrovascular disease	FEESST	Tactile	Tip of endoscope	Cough (presence or absence), swallow, LAR	Fibreoptic endoscopic dysphagia severity scale (FEDSS) score of 1
Borders et al. [61]	2020	Mixed aetiologies	FEESST	Tactile	Tip of endoscope	LAR	LAR
Ishibashi & Fujishima [62]	2012	Cerebrovascular disease	FEESST	Tactile	Tip of endoscope	LAR	LAR
Martin et al. [63]	1996	Cerebrovascular disease	FEESST	Tactile	Air pulse	Raising hand when stimulus is detected	LAR at < 3.5 mmHg air pulse pressure
Aviv et al. [64]	1998	Mixed aetiologies	FEESST	Tactile	Air pulse	Raising hand when stimulus is detected	Raising hand within 2 s of stimulus presentation
Kaneoka et al. [41]	2015	Mixed aetiologies	FEESST	Tactile	Tip of endoscope	Press buzzer to indicate detection of stimulus, facial expressions	Press buzzer to indicate detection of stimulus
Aviv et al. [7]	1997	Cerebrovascular disease	FEESST	Tactile	Air pulse	Raising hand when stimulus is detected	Detection response
Martin et al. [43]	1999	Mixed aetiologies	FEESST	Tactile	Air pulse	Raising hand when stimulus is detected	Raising hand within 2 s of stimulus presentation
Giraldo-Cadavid et al. [17]	2016	Mixed aetiologies	Gag Reflex Testing	Tactile	Air pulse	NR	NR
Giraldo-Cadavid et al. [58]	2018	Mixed aetiologies	Gag Reflex Testing	Tactile	Air pulse	NR	NR
Andrews & Pillay [65]	2017	N/A	Gag Reflex Testing	NR	NR	NR	NR
Bateman et al. [11]	2007	N/A	Gag Reflex Testing	NR	NR	NR	NR
Kambayashi et al. [18]	2021	N/A	Gag Reflex Testing	Tactile	‘tactile stimulation’	NR	NR
Cunha et al. [66]	2020	Head and neck cancer	Taste Testing	Gustatory	Taste strips	NR	9 correct taste answers
Pauloski & Nasir [67]	2016	Mixed aetiologies	Taste Testing	Gustatory	Sweet (sucrose) bolus / Sour (citric acid) bolus	Recognition of taste or its absence	3 correct taste answers
Bateman et al. [11]	2007	N/A	“Sensory function” (p. 179)	NR	NR	NR	NR
Andrews & Pillay [65]	2017	N/A	‘Sensory function (taste/smell/touch)’ (p. 5)	NR	NR	NR	NR

CRT = Cough Reflex Testing; UTC = Urge to Cough; FEEST = Flexible endoscopic evaluation of swallowing with sensory testing; LAR = laryngeal adductor reflex

### FEESST

FEESST was described in 30 studies (56%). Two methods were identified in the literature: the elicitation of the LAR and the perceptual method.

### FEESST: Elicitation of the LAR

The most common method, described in 90% of FEESST studies, involved elicitation of the LAR. The LAR was elicited using an air pulse stimulus (*n* = 23) and by touching the arytenoids with the tip of the endoscope (*n* = 5). When reported, different air pulse pressures were used across studies, ranging from 0 to 10 mmHg for 50ms duration. Nine studies (45%) did not report the pressures assessed. The air pulse detection thresholds for normal sensation (< 4 mmHg), moderate impairment (4–6 mmHg) and severe impairment (> 6–7 mmHg) were similar across studies.

### FEESST: Perceptual Method

Evaluating the perception of a sensory stimulus during FEESST was described in six studies. Air pulses of 50ms duration were used as the sensory stimulus in four studies, with pressures ranging from 0 to 15 mmHg across studies. The tip of the endoscope was used as the sensory stimulus in the remaining two studies. Participants indicated their perception of the sensory stimulus by raising their hand or pressing a buzzer.

### CRT

CRT was reported in twenty-two (40%) of the included studies. Four different stimuli were used across studies: capsaicin aerosol (*n* = 9), citric acid aerosol (*n* = 9), tartaric acid (*n* = 2), and air pulse (*n* = 1). One study did not report the stimulus used [18]. Different stimuli, concentrations of aerosols (Table 2) and methods of CRT (e.g., single dose, dose response, tidal inhalation, single breath inhalation) were used across studies, even within studies that used the same aerosol.

**Table 2:** Methods of CRT reported in the dysphagia literature

Stimulus	Author	Year	Stimulus description
Air pulse	Giraldo-Cadavid et al. [17]	2016	Air pulses
Capsaicin aerosol	Yiu et al. [19]	2019	50, 100, 200 µM
	Borders et al. [20]	2022	50, 100, 200 µM
	Troche et al. [21]	2014	50, 100, 200 µM
	Tabor-Grey et al. [25]	2021	50, 100, 150 and 200 µM
	Troche et al. [26]	2016	50, 100, 150 and 200 µM
	Curtis & Troche [27]	2020	10, 25, 50, 100, 500 µM
	Borders et al. [23]	2021	10, 25, 50, 100, 500 µM
	Silverman et al. [24]	2016	50, 100, 200 and 500 µM
	Smith & Wiles [22]	1998	0.000007–0.002 mol/L
Citric Acid aerosol	Wakasugi et al. [28]	2008	1% w/v citric acid-physiological saline solution
	Wakasugi et al. [30]	2014	1% w/v citric acid-physiological saline solution
	Guille´n-Sola et al. [31]	2015	1% w/v citric acid-physiological saline solution
	Lee et al. [32]	2014	1% w/v citric acid-physiological saline solution
	Sato et al. [33]	2012	1% w/v citric acid-physiological saline solution
	Sakai et al. [29]	2016	10% w/v citric acid
	Kambayashi et al. [18]	2021	NR
	Sohn et al. [34]	2018	0.28 mol/L (5% citric acid) diluted in 0.9% sodium chloride
	Choi et al. [36]	2017	0.28 mol/L (5% citric acid) diluted in 0.9% sodium chloride
	Dallal-York et al. [35]	2023	citric acid diluted in 0.9% sodium chloride
Tartaric acid aerosol	Fujiwara et al. [37]	2017	20% solution of prescription-grade l-tartaric acid dissolved in 2 mL of sterile normal saline
	Ohno et al. [83]	2021	10% tartaric acid solution

### Gag Reflex Testing

Gag reflex testing was reported in five (9%) of the included studies [11, 17, 18, 57, 64]. Two papers, by the same author, discussed gag reflex testing during FEESST [17, 57]. In these studies, the gag reflex was elicited from “the lateral wall of the pharynx, at a point lateral to the epiglottis” [67]. The remaining studies were survey studies of speech pathology practice patterns. The gag reflex was listed as a component of the clinical swallowing evaluation [11, 18, 64]. Its use varied from 15 to 50% in these studies. Precise methods of gag reflex testing were not described in any of these studies.

### Taste Testing

Taste testing was reported in two studies [65, 66]. Different stimuli were used across the studies. One study used taste strips comprising different concentrations of salty, sweet, and sour stimuli that were placed on the tongue [65]. Another study used 0.005–1 mol/L citric acid and 0.005–1 mol/L sucrose boluses. Droplets of each were placed on the tongue using a syringe and participants were asked to identify the taste. The criteria for adequate gustatory function differed in both studies. For the taste strips method [65], nine correct answers were required. For the citric acid and sucrose boluses, correct recognition of three consecutive concentrations for each stimulus was required [66].

### Tests of Sensory Function

Two of the included studies described non-specific tests of sensory function [11, 64]. These included a ‘test of sensory function’ [11] and a tool for ‘sensory function (taste/smell/touch)’ [64]. Both were survey studies that evaluated speech pathology practice patterns. Precise methods of the sensory tests were not described in either study.

## Discussion

This study summarised and appraised methods of upper airway sensory testing in the dysphagia literature to inform clinical practice and future research directions. Four upper airway sensory tests were identified in the dysphagia literature. These included FEESST, CRT, gag reflex testing and gustatory testing. Two studies also described indeterminate tests of sensory function.

FEESST and CRT were the most common methods of upper airway sensory testing in the dysphagia literature. Both tests evaluate laryngeal sensation and the integrity of airway protective and clearance mechanisms through different mechanisms. CRT uses chemical aerosols (citric acid, tartaric acid, capsaicin) to induce cough and airway clearance mechanisms. Whereas FEESST uses tactile stimuli (air pulse, touch of the endoscope). The precise location at which sensation is assessed is different for each test. FEESST evaluates supraglottic sensation from the arytenoids supplied by the superior laryngeal nerve, whereas CRT evaluates supra- and sub-glottic sensation supplied by the superior and recurrent laryngeal nerves respectively, through aerosol inhalation. Perception of upper airway sensation can also be evaluated for FEESST and CRT. Individuals can indicate their perception of the sensory stimulus by raising their hand or pressing a buzzer during FEESST or by rating the level of perceived stimulus intensity using the UtC scale during CRT. Assessment of both the presence or absence of motor responses (i.e., cough or LAR), and individuals’ perception of sensory stimuli is valuable. This is because cough and airway clearance mechanisms are elicited under varying levels of cortical control [68, 69], and blunted perception to cough-inducing stimuli can reduce airway clearance capacity [21, 70]. Additionally, upregulation of individuals’ perception to cough-inducing stimuli is also a promising avenue for rehabilitation of airway protection and clearance mechanisms [71, 72].

Discontinuation of FEESST instrumentation for air pulse testing may have increased the use of CRT in dysphagia research and clinical practice. However, a major limitation of CRT is the lack of standardised methods [73]. Numerous studies have validated CRT against instrumental assessment for identifying risk of laryngeal sensory deficits resulting in silent aspiration [30, 32, 74–77]. However, the findings of this study highlight that few studies in the dysphagia literature are using validated methods of CRT [78]. Consensus on the optimal methods of CRT in dysphagia research and clinical practice would be valuable in advancing the field.

Gag reflex testing was reported in five studies in the literature [11, 17, 18, 57, 64]. Three of these studies were clinician questionnaires, which aimed to investigate current clinical practices in dysphagia assessment [11, 18, 64]. The remaining two studies performed the gag reflex test as an additional measure of pharyngeal sensation during FEESST [17, 57]. The gag reflex test evaluates the integrity of the glossopharyngeal nerve (CN IX) and pharyngeal muscles. A key limitation of this test is the high number of individuals (up to 75%) with an absent gag response, especially among the elderly [79]. This makes it difficult to draw meaningful conclusions about pharyngeal sensory integrity if a gag response is absent. Additionally, the administration of the test can vary across clinicians, including the site of elicitation (faucial arches, posterior pharyngeal wall, back of tongue) and the amount of pressure applied. The results of this study indicated that the clinical use of this test differed across geographical locations and professions. Few clinicians (15%) used the gag reflex test as part of their clinical swallowing evaluation in the UK and Ireland [11]. Whereas one third of clinicians used the test in South Africa. A higher number (~ 50%) of otolaryngologists used the gag reflex test as part of their clinical dysphagia assessment in Japan.

Gustatory testing was used in two of the included studies, both of which used different methodologies. Taste is important for swallowing function, with improved swallowing biomechanics observed with sweet and sweet-sour boluses compared to unflavoured [80]. We also know that blunted taste (ageusia) can have adverse effects on quality of life and nutritional status [81]. Assessment of taste from the two included studies was relative low cost and clinically applicable, and therefore could be easily implemented as part of the clinical swallowing evaluation. Consensus on the optimal methods for taste testing would be valuable in clinical dysphagia practice and research.

Two studies described tests of “sensory function” without providing details on what this constituted. Both studies were surveys of clinical dysphagia practices and therefore likely related to sensory testing as part of the clinical swallowing evaluation. However, it was unclear what types of sensory function was assessed and how it was assessed from these studies. Nevertheless, it suggests that sensory testing comprises part of the clinical swallowing evaluation among some clinicians. Consensus on the optimal methods of sensory testing during a clinical swallowing evaluation would be beneficial and is the focus of an ongoing study.

### Future Directions

There is a need for standardized methods of upper airway sensory testing in dysphagia research and clinical practice. Upper airway sensory testing is performed in other patient populations, for example, in individuals with obstructive sleep apnea (OSA). Seven categories of upper airway sensory testing, including olfactory, gustatory, chemical, tactile, vibratory, and thermal sensation [82] are assessed. These tests would be highly applicable to a dysphagia patient population. Developing a valid and reliable protocol of upper airway sensory testing for dysphagia research and clinical practice is a top priority and the focus of ongoing studies in our research lab.

### Limitations

This review focused on upper airway sensory testing in the adult dysphagia population therefore, methods of upper airway sensory testing in children with dysphagia, healthy individuals and/or other patient populations not presenting with dysphagia were excluded. Our review was limited to studies in English. There were challenges in synthesizing and comparing different methods of upper airway sensory testing due to the difficulty in finding studies with comparing designs.

## Conclusion

This review provides a comprehensive overview of methods of upper airway sensory testing in the dysphagia literature for dysphagia clinicians and researchers. Four methods of upper airway sensory testing were reported in the dysphagia literature: FEESST, CRT, gag reflex testing and taste testing. The results indicate a need to develop a valid and reliable upper airway sensory testing protocol for individuals with dysphagia to improve assessment and targeted treatments.

## Electronic Supplementary Material

Below is the link to the electronic supplementary material.

Supplementary Material 1

## Data Availability

All data collected and analysed for this study is included in the publication.
